# Digital Record for Removable Denture Patients

**DOI:** 10.1155/2023/5712978

**Published:** 2023-01-19

**Authors:** G Krishna Teja, BL Rao, TSV Satyanarayana, TLG Sravanthi, D Padmini, CD Saikumar

**Affiliations:** Department of Prosthodontics, Lenora Institute of Dental Sciences, NTR University, Rajanagaram, Andhra Pradesh, India

## Abstract

Denture marking is required for forensic and social reasons in the event that patients must be identified individually. The majority of surface marking and inclusion techniques are costly and time-consuming, and do not allow for the incorporation of large amounts of data. Near-field communication (NFC) is a popular wireless technology that allows you to transfer data between two devices that are in close proximity to each other. This tag features an internal memory that can be used to store patient information. Incorporating this tag into a denture can make things handy for storage of information digitally.

## 1. Introduction

According to most international dental societies and forensic odontologists, every denture should be labelled. Labelling dentures is, in fact, legally mandated in a number of countries and jurisdictions throughout the United States. A dentist is expected to keep complete dental records of his patients as a member of the profession. Documenting the identity of dentures is one example [1]. Denture identification provides critical clues in recognizing the denture-wearer and is especially vital in a forensic scenario, bringing the case to a successful conclusion. If an edentulous individual is involved in a disfiguring accident, having his dentures uniquely tagged or labelled makes it easier to identify him. The denture is usually identified by a small, discrete identifying code inserted in the denture base [2]. Denture labelling serves two purposes: it aids in the identification of edentulous people, both living and deceased, as well as the return of a misplaced denture. When incorporated into a denture, denture markers must be biologically inert, affordable, simple and quick to apply, retrievable after an accident, acid resistant, and able to withstand high temperatures [3]. The marking must also be aesthetically pleasing, visible (readable), and long-lasting without jeopardizing the prosthesis's strength. The marking must also be durable and resistant to conventional cleaning and disinfection agents. As a result, the posterior regions of the lingual flange and the palate are recommended for marking [4].

Various methods of denture marking have been published in the literature over the years. Micro-labels [5], AADHAR numbers [6], lenticular cards [7], radio frequency identification tags [8], photographic methods [9], and other surface marking and inclusion techniques are among them. All of these techniques have their own shortcomings, such as the fact that they are time-consuming, may not be aesthetically pleasing, and do not allow for the incorporation of a large amount of data. This study describes a simple, rapid, and cost-effective way for identifying individuals and keeping track of outpatient records.

## 2. Case Report

A 53-year-old male patient reported to the Department of Prosthodontics, Lenora Institute of Dental Sciences, Rajanagaram, Andhra Pradesh, India. The patient presented with a chief complaint of complete edentulousness and wants to get his teeth replaced. On clinical examination, there were moderately resorbed maxillary and mandibular ridges. A conventional complete denture was planned for the patient. Due to the progressing age of the patient, the patient had difficulty in memorizing things and frequently lost his outpatient record. Therefore, we opted for a unique denture identification system, which not only can present the patient's name, age, gender, and address but also can maintain a record of the patients previously underwent and ongoing treatment details in a systematic manner.

**Figure 1:**
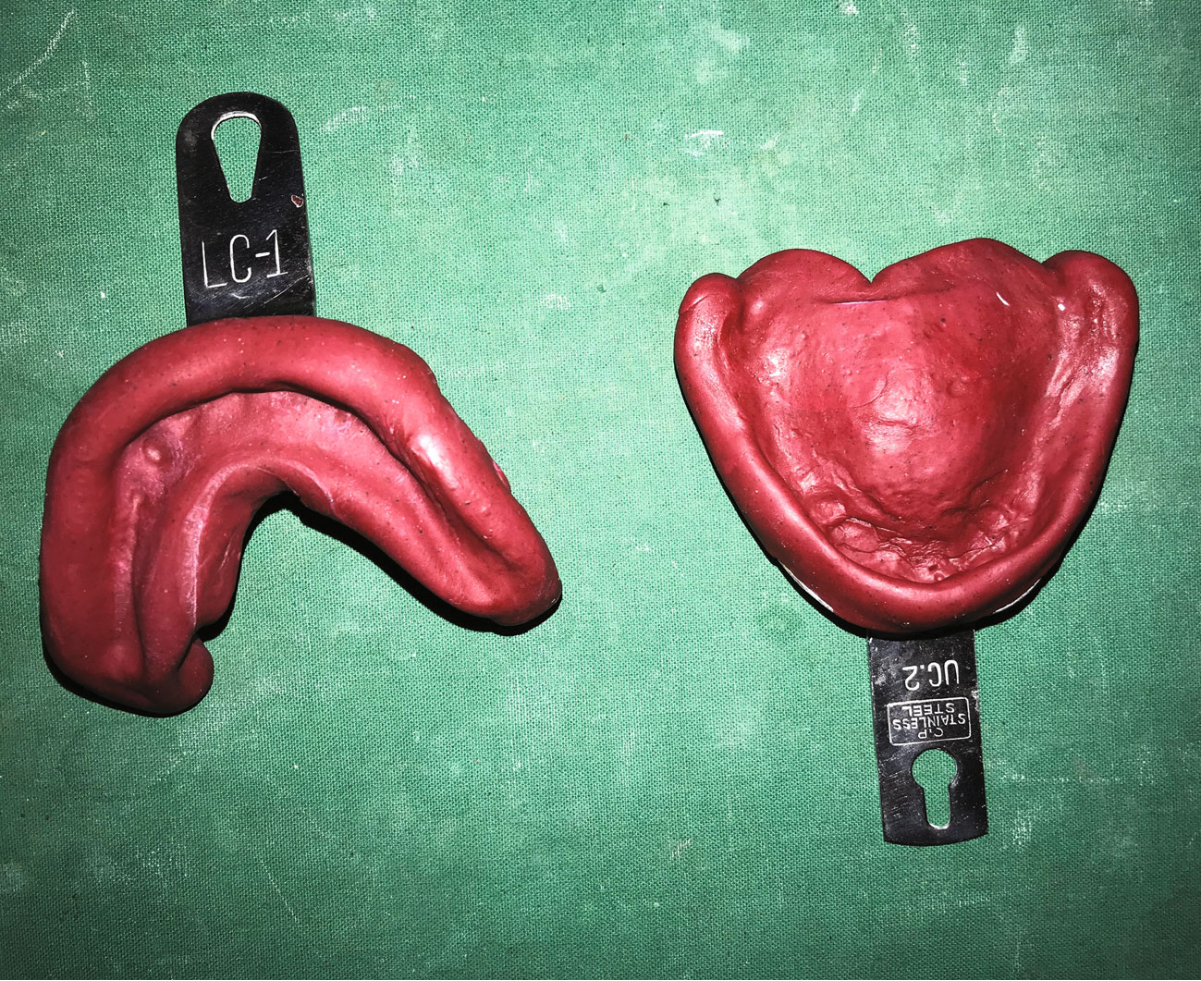
Primary impression.

A primary impression of the patient's edentulous ridges was made with impression compound (Figure 1).

Primary casts were poured in Plaster of Paris, and a wax spacer was fabricated over the casts.

**Figure 2:**
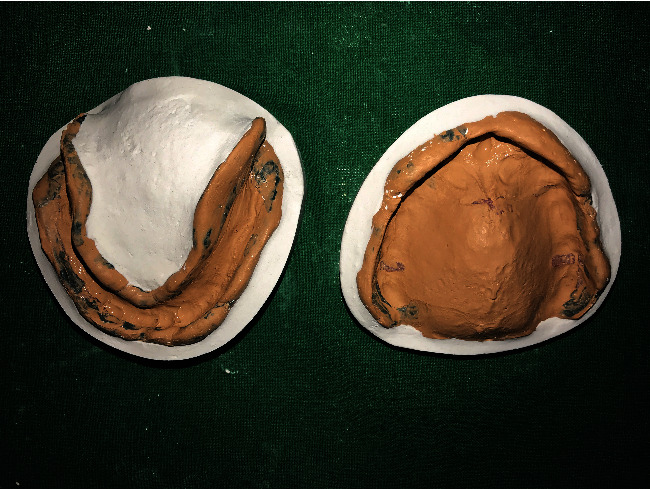
Secondary impression.

Secondary impression (Figure 2) was made using zinc oxide eugenol impression paste.

Master casts were poured in dental stone, and occlusal rims were fabricated over the record bases.

**Figure 3:**
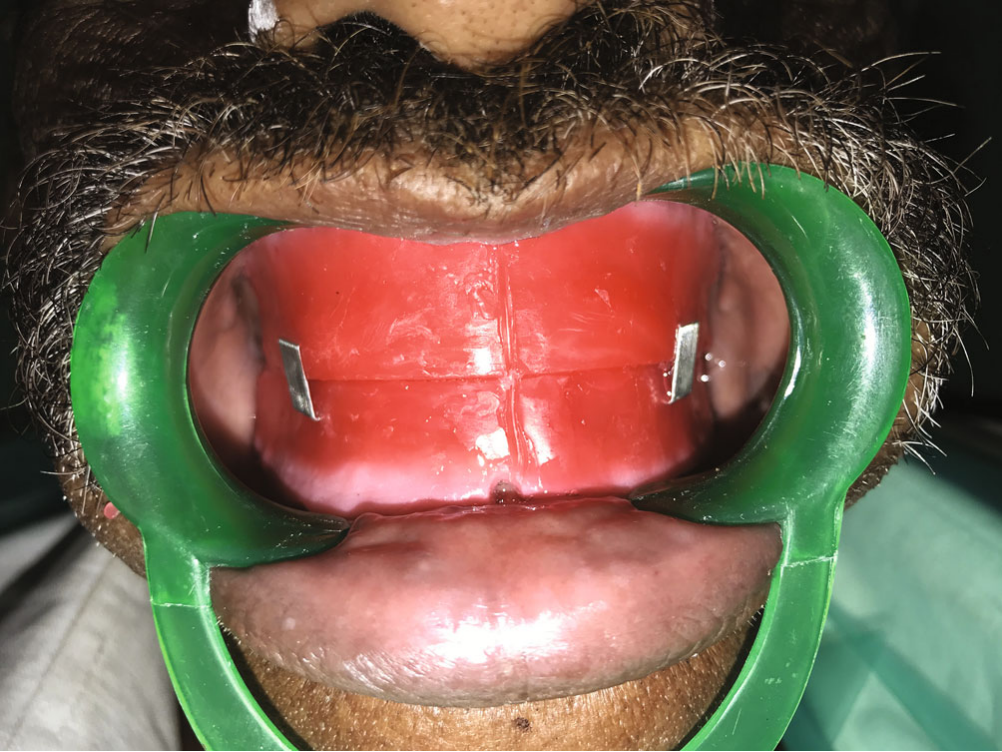
Jaw relation.

Jaw relation was done, and teeth arrangement was done using semi-anatomic teeth (Figure 3).

**Figure 4:**
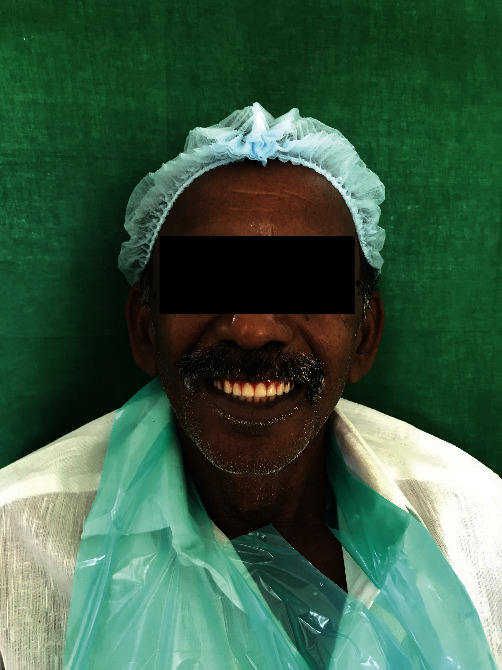
Try-in.

Try-in was done (Figure 4).

**Figure 5:**
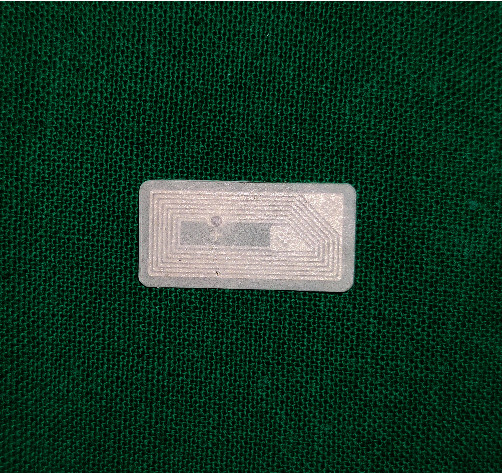
NFC tag.

**Figure 6:**
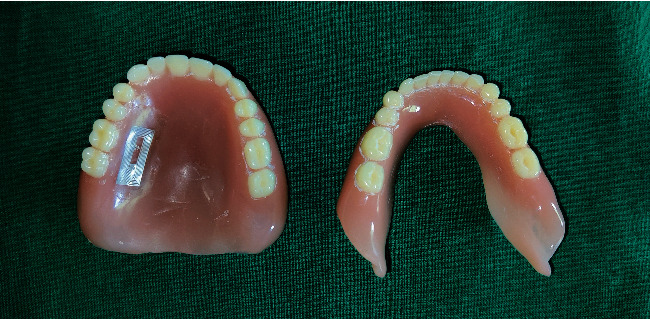
NFC tag incorporated into maxillary denture.

**Figure 7:**
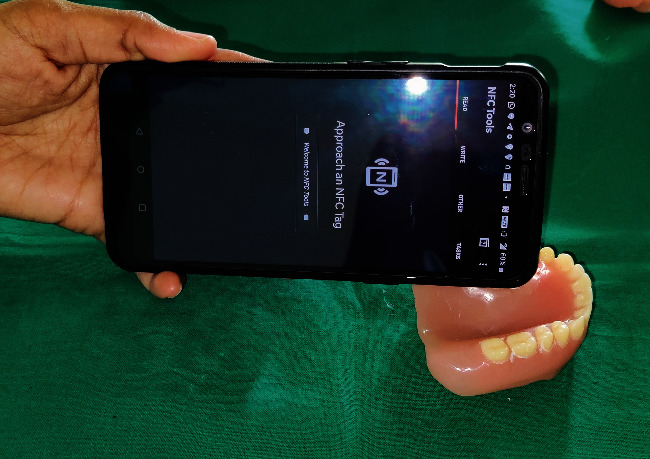
Approaching NFC tag.

**Figure 8:**
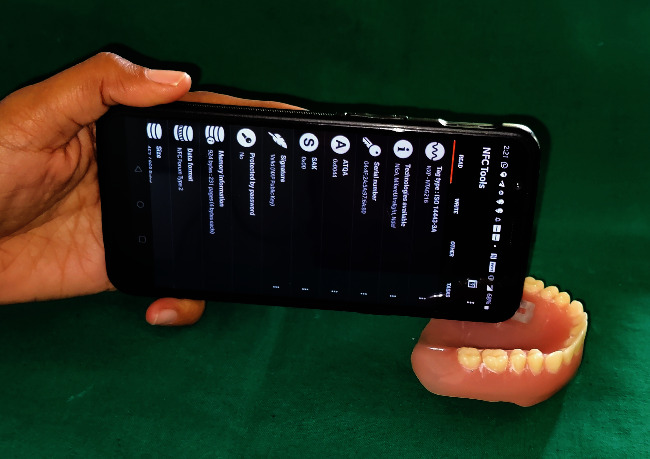
NFC tag detected.

**Figure 9:**
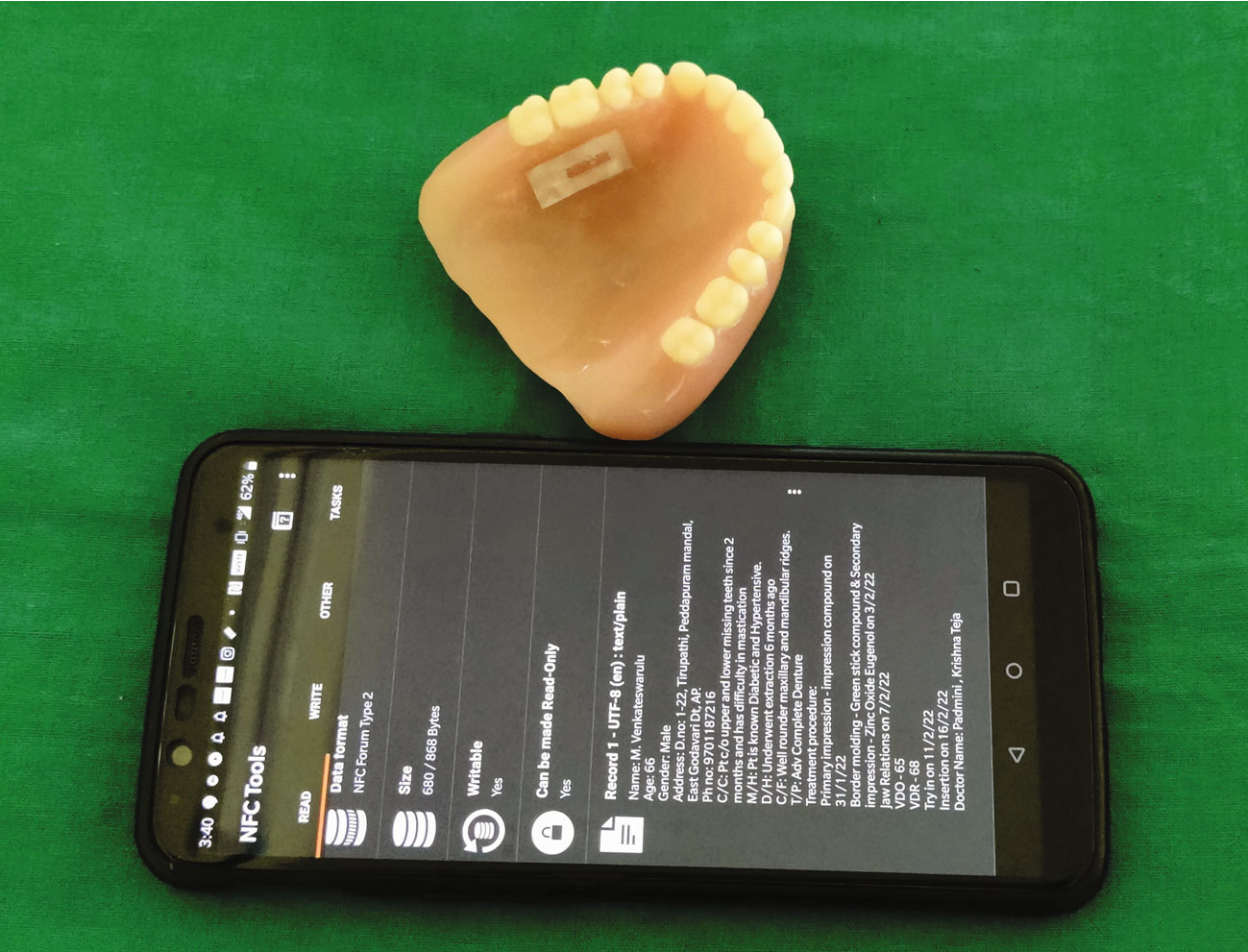
Secured patient information in NFC tag.

- Patient was satisfied with the aesthetics.
- Flasking and dewaxing were done, and packing was done with heat cure polymethyl methacrylate material.
- Later, the flasks were placed in an acrylizer, and curing was done.
- The dentures were retrieved, finished, and polished.
- An outline of the near-field communication (NFC) tag was marked over the hard palate region of the denture, and a trough was created to a depth of 1 mm using tungsten carbide bur over the marked outline.
- The NFC tag (Figure 5) was placed in the trough, and self-cure clear acrylic was used to seal the trough to the normal contours of the hard palate (Figure 6).

- Dentures were given to the patient.
- Patient had a smartphone, which had a built-in feature of NFC.
- We installed NFC tools application in his smartphone through Google play store.
- Through the application, we have entered the patient details like patient's name, age, gender, address, emergency contact number, and ongoing treatment details right from the primary impression to the insertion with date (Figure 7).

- The NFC tag works simply by scanning the tag through the NFC tools application (Figure 8).

- All the patient details were visible from the NFC tag, and further, the information can be protected through a password, and it is highly secured (Figure 9).

- The tag can resist fire, water, and acidic insults.
- In future, whenever the patient lost his outpatient record or in any catastrophic situations, the details that are provided in the tag will be highly beneficial.

## 3. Discussion

Smartphones have evolved into an additional organ for every human, serving not only to make calls but also to perform a variety of vital actions in our everyday lives. Because of the rapid improvement of smartphone technology, a slew of novel choices have emerged, one of which is NFC. When both devices are NFC equipped, it can transfer data from one device to other. For hospitalized patients, those in long-term care facilities, forensic purposes, and denture identification systems are useful. Denture labelling is becoming increasingly significant as regulatory authorities recommend that all prostheses be labelled with an identification scheme [10]. Only Sweden and Iceland have laws governing denture marking. In 1986, Sweden's “National Board of Health and Welfare,” which oversees the country's health-care system, passed legislation requiring all dentists to follow the following guidelines: “The patient shall always be provided the chance to have his or her dentures marked with a personal number.” In addition to the foregoing, the dentist should always explain the benefits of denture marking to the patient in a straightforward and motivating manner [11, 12]. Denture marking is required in 21 states in the United States; however, it is only done at the request of the patient in New York. Long-term care facilities in certain states are required to mark their dentures, and the army is required to do so.

Ling [5] used a microlabel procedure that uses a personal computer, printer, plain paper copier, and transparency film for photocopying and stated that the procedure is easy to use and is cost-effective, because it uses equipment that exists in any office or institution. Colvenkar [7] proposed the method to include a lenticular identification card, which is simple and cheap, and can store a large amount of information, thus allowing quick identification of the denture wearer. Mahoorkar and Jain [13] used patient's unique identification number and barcode printed in the patient's Aadhaar card issued by Unique Identification Authority of India as denture markers. Bathala et al. [14] proclaimed that if dentists/prosthodontists and laboratories keep correct records and mark/labelled all of their patients' prostheses, “prosthodontics” will truly become a forensic dentistry weapon. Venkateshwaran et al. [15] declared that the 2D barcode denture marking system enables instant identification of the denture-wearer, holds loads of information including a photograph, and is cost-effective, thus making its use feasible in a developing country like India.

Outpatient records, whether computerized or handwritten, are critical to ensuring patient continuity of care. Outpatient records are vital for health professionals who are defending a complaint or clinical negligence claim. They give us a glimpse into the clinical judgement that was being used at the time. A complete, up-to-date, and accurate outpatient record can make a significant difference in the outcome [13]. NFC is a popular wireless technology that allows you to transfer data between two devices that are in close proximity to each other. The fundamental benefit of incorporating the NFC tag inside a denture is that no additional equipment is required to scan the tag. There are a variety of apps available in the app store that can read and/or write NFC tags. This tag features an internal memory that can be used to store patient information. These tags are water-resistant and can easily resist the curing temperatures of a denture. Moreover, the information that is stored in the tag can be secured using a password. The tag is rewritable, and the data can be edited as many times we want. The only short coming of this technique is that the data that can be written into this tag is limited. As technology advances, there is a better potential of developing tags with higher memory capacity if this technique is well-accepted by people.

## 4. Conclusion

A simple and cost-effective means of denture identification and record maintenance, based on the use of NFC tag, is described here. The NFC tag can store a sufficient amount of information that can be readily accessible while identifying a person in a catastrophic situation or to view the patient's previous treatment modality. This technique can be used for both complete and removable partial dentures.

## References

[1] Alexander P. M., Taylor J. A., Szuster F. S., Brown K. A. (1998). An assessment of attitudes to, and extent of, the practice of denture marking in South Australia. *Australian Dental Journal*.

[2] Datta P., Sood S. (2010). The various methods and benefits of denture labeling. *Journal of Forensic Dental Sciences*.

[3] Borrman H. I., DiZinno J. A., Wasén J., René N. (1999). On denture marking. *The Journal of Forensic Odonto-Stomatology*.

[4] Wilson H. J., Mansfield M. A., Heath J. R., Spence D. (1987). *Dental Technology and Materials for Students*.

[5] Ling B. C. (1998). Computer-printer denture microlabeling system. *The Journal of Prosthetic Dentistry*.

[6] Pathak C., Pawah S., Sikri A., Rao I. (2018). Unique denture identification system for all Indian nationals. *Contemporary Clinical Dentistry*.

[7] Colvenkar S. S. (2010). Lenticular card: a new method for denture identification. *Indian Journal of Dental Research*.

[8] Nuzzolese E., Marcario V., Di Vella G. (2010). Incorporation of radio frequency identification tag in dentures to facilitate recognition and forensic human identification. *The Open Dentistry Journal*.

[9] Krishna teja G. V. R. S. R., Rao B. L., Singh N. K., Sravanthi T. L. G., Monika P. K., Aditya K. (2021). Being unconventional in complete dentures: a review. *Journal of Prosthodontics Dentistry*.

[10] McGivney G. P., Averill D. C. (1991). Marking of removable dental prosthesis. *Manual of Forensic Odontology*.

[11] Stenberg I., Borrman H. I. (1998). Dental condition and identification marking of dentures in homes for the elderly in Göteborg, Sweden. *Journal of Forensic Odonto-Stomatology*.

[12] Swedish National Board of Health and Welfare SOSFS

[13] Mahoorkar S., Jain A. (2013). Denture identification using unique identification authority of India barcode. *Journal of Forensic Dental Sciences*.

[14] Bathala L. R., Rachuri N. K., Rayapati S. R., Kondaka S. (2016). Prosthodontics an “Arsenal” in forensic dentistry. *Journal of Forensic Dental Sciences*.

[15] Venkateshwaran R., Manoharan P. S., Karthigeyan S., Konchada J., Ramaswamy M., Janardhanam D. (2014). Denture markers: a comparison. *Journal of Indian Academy of Dental Specialist Researchers*.

